# Determination of the apoptotic index in osteosarcoma tissue and its relationship with patients prognosis

**DOI:** 10.1186/1475-2867-13-56

**Published:** 2013-06-04

**Authors:** Xing Wu, Biao Cheng, Zheng-dong Cai, Lie-ming Lou

**Affiliations:** 1 Department of Orthopaedics, Shanghai tenth People’s Hospital, Tongji University School of Medicine, No.301 Middle Yanchang Road, Shanghai, 200072, China

**Keywords:** Osteosarcoma, Apoptosis, Biomarker, Prognosis

## Abstract

**Background:**

Nowadays it remains a controversial issue whether a correlation exists between the apoptosis rate of tumor tissue and the prognosis of the patients. We aimed to explore the prognostic significance of apoptosis index of human osteosarcoma tissue.

**Methods:**

The technique of terminal DNA breakpoints in situ 3 - hydroxy end labeling (TUNEL) was used to detect and analysis apoptosis index in 56 osteosarcoma specimens. The relationships between apoptosis index of tumor tissue and long term survival of patients as well as pathologic classification, tumor clinical stages, tumor size and level of serum alkaline phosphatase were analyzed.

**Results:**

Our studies showed the cases with high apoptosis index had significantly longer survival time. Apoptosis index in osteosarcoma tissue was correlated with tumor size and level of serum alkaline phosphatase but not with pathologic classifications and clinical stages of tumor.

**Conclusion:**

Our results demonstrated that apoptosis index of osteosarcoma tissue combined with serum alkaline phosphatase could used as valid indicators to predicate the malignant level and prognosis of osteosarcoma cases, which would contribute to enhance efficacy of clinical treatments for osteosarcoma.

## Introduction

Osteosarcoma is the most frequent highly malignant bone-tumor with a peak manifestation during the second and third decade of life. Survival rates in osteosarcoma have improved considerably from 20 to 65% since the 1980s with the advent of multiagent chemotherapy [1,2]. Further improvement in survival has not been achieved owing to lack of well-validated prognostic markers and the problem of non-response to chemotherapy [3,4]. The identification of patients with a bad response to therapy at the time of diagnosis would facilitate already a preoperative stratification of chemotherapy or a more aggressive regime to increase survival.

It’s the recent realization of malignant tumors may grow due to not only limitless proliferation but also athanasy of tumor cells that provides absolute new directions in basic and clinical tumor research. Thus apoptosis, namely the programmed cell death, is among the spotlights of academic area of tumor [5,6], which has been found playing an important role during the occurrence and development of tumors [7,8]. However, to this day it remains a controversial issue whether the correlation of apoptosis rate with the degree of malignancy and prognosis of the patients exist [9]. Furthermore, what about the relationship between apoptosis and osteosarcoma? Few studies yet have been undertaken to explore the predictive value of apoptosis on the therapy for osteosarcoma cases.

We hypothesized that it were the unstable experimental techniques for detecting different phase of apoptosis of tumor cells that had influenced the results of apoptotic rates. In our study apoptotic index (AI) in paraffin sections of osteosarcoma tissue was detected and calculated through technique of terminal deoxynucleotidyl transferase (TdT)-mediated dUTP-digoxigenin nick end labeling (TUNEL). The relationships between apoptotic index of osteosarcoma tissue and the long-term survival of patients and interrelated influencing factors were analyzed.

## Materials and methods

### Patients

Paraffin-embedded tissues were obtained from 56 patients with limb osteosarcoma who had undergone limb salvage surgery by the same senior surgeon between 1994 and 2006. The diagnosis of patients was confirmed by a pathologist. Serial sections of 4μm thickness were used for H&E staining and immunohistochemistry. The group included 26 cases of osteoblastic type, 16 cases of fibroblastic type, 10 cases of chondroblastic type and 4 cases of mixed type according to pathologic classification [10]. There were 36 males and 20 females aged from 14 to 37 years in the group. All the 56 patients underwent the same regimes of neoadjuvant chemotherapy (high-dose methotrexate adriamycin and cisplatin). Patients were followed up for 2 to 8.5 years (median 5.8 years). Our study was approved by an Investigational Review Board and followed principle in the Declaration of Helsinki.

### Terminal DNA breakpoints in situ 3 - hydroxy end labeling (TUNEL)

The TUNEL staining kit was from Beohringer Mannheim GmbH. Staining precedures were as following: (1) The tissue sections were deparaffinized by immersing slides in xylene, and rehydrated by sequentially immersing the slides through graded ethanol washes. (2)The activity of endogenous peroxidase was quenched by adding 0.3% H_2_O_2_ for 30 minutes and then washed in 10 mmol/l PBS (pH 7.4), 3 times for 5 min each. (3)The sections were digested with 20 μg/ml Proteinase K for 25 minutes at room temperature, and washed in 10 mmol/l PBS (pH 7.4), 3 times for 5 min each. (4)The TUNEL reaction mixture was added to each section followed by incubating in a humid chamber for 60 min at 37°C. (5) They were then washed in 10 mmol/l PBS (pH 7.4), 3 times for 5 min each, and incubated with peroxidase conjugated anti-fluorescein antibody in a humid chamber for 30 min. at 37°C. Then they were washed in 10 mmol/l PBS (pH 7.4), 3 times for 5 min each. (6) The tissue sections were stained with DAB for 10 min, counterstained with hematoxylin, dehydrated, cleared in xylene and cover slipped.

### Microscope observation

**Figure 1:**
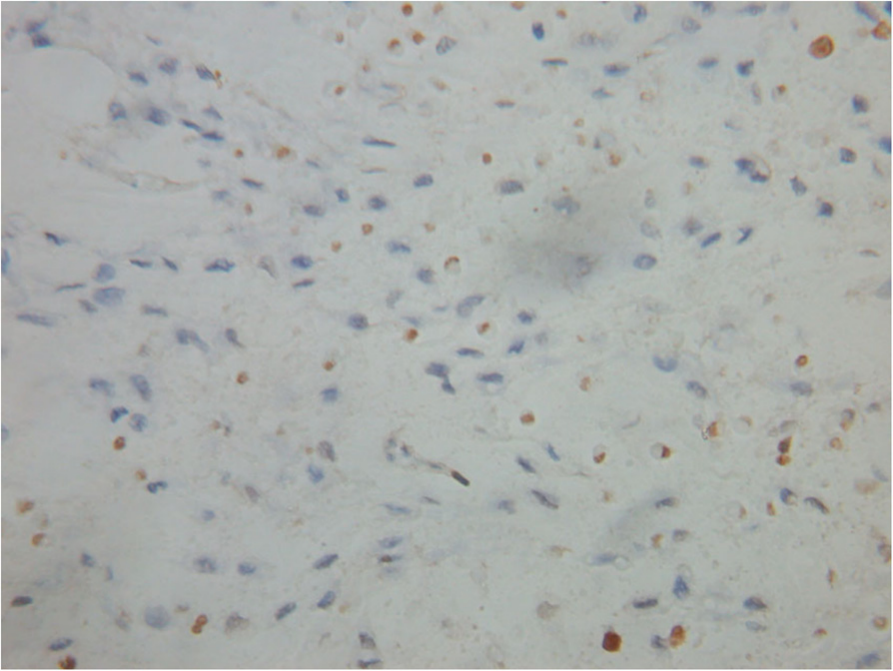
Positive expression of apoptotic osteosarcoma cells (Tunel staining ×400).

**Figure 2:**
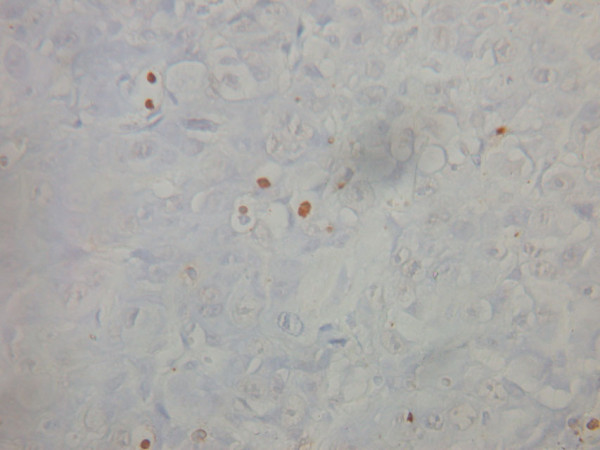
Apoptotic bodies (Tunel staining ×400).

Morphological features of apoptotic cells: TUNEL staining was able to distinguish apoptotic cells and apoptotic bodies with surrounding cells. Proteins were located in the nuclei, were brown in color, with condensed nucleoplasm and in close proximity to the nuclear membrane, or were present as diffuse staining. In addition, apoptotic bodies were also found in the sections (Figures 1 and 2). The apoptotic index of the cases in this group was 0% ~ 17.8% (median 8.45%), the positive rate was 53.6% (30/56).

### Determination of the apoptotic Index (AI)

The apoptotic index was determined by counting the percentage of positive cells from at least 500 neoplastic nuclei at 400× magnification. For each slide, five most obvious positive staining fields were examined. Based on the AI, cells were divided into four grades: grade I: apoptotic cells not detected or AI <1% correspondent to negative cells, (−); grade II: AI< 5% of cells were (+); grade III: 5%~10% of cells were (++); grade IV: >10% of cells were (+++).

### Statistical analysis

A statistical software package (SPSS 10.0) was used to carry out the statistical analysis. The correlation analysis of the rank correlation coefficient between two groups was carried out using the Wilcoxon W test. The correlation analysis of the rank correlation coefficient between multiple independent samples was carried out using the Kruskal-Wallis tests.

## Results

### Relationship between gross tumor volume and AI of osteosarcoma

Our study showed that the grade of apoptotic index in osteosarcoma tissue of gross tumor volume ≥ 10cm was significantly different compared with that of gross tumor volume < 10 cm (Table 1).

**Table 1:** Relationship between gross tumor volume and AI of osteosarcoma

Tumor volume *	**A I (cases)**			
	**-**	**+**	**++**	**+++**
≥10 cm	4	6	8	6
<10 cm	22	6	4	0

*:Wilcoxon Rank test analysis : Z=−2.994 , P<0.01.

### Relationship between levels of patient’s serum alkaline phosphatase and AI of osteosarcoma

The normal value of people’s serum alkaline phosphatase (ALP) ranges from 15 to115 IU/L. According to literature reported [11] we defined ALP >200 IU/L as abnormity in children and adolescent phase concerning elevation of ALP value at physiological skeleton growth period. Wilcoxon W rank test revealed the grade of AI in the ALP elevated group was significantly different compared with that in the normal ALP group (Table 2).

**Table 2:** Relationship between levels of serum ALP and AI of osteosarcoma

ALP*	**AI**			
	**-**	**+**	**++**	**+++**
Elevated	18	4	4	0
Normal	8	8	8	6

* Wilcoxon W rank test: Z=−2.332, P<0.05.

### Relationship between AI of osteosarcoma and the long term survival of patients

The grade of AI in patients with long term survival >5 years was statistically higher compared with that in patients with long term survival ≤ 5 years, and the results demonstrated that the grade of AI in the long term survival of patients with osteosarcoma was significantly different compared with that in the group with survival time less than 5 years (Table 3).

**Table 3:** Relationship between AI of osteosarcoma and the long term survival of patients

Long term survival*	**AI**			
	**-**	**+**	**++**	**+++**
>5 years	3	6	8	5
≤5years	23	6	4	1

* Wilcoxon W rank test: Z= −2.407,P<0.05.

## Discussion

The concept of apoptosis has a far-reaching impact on cancer awareness and research. Although apoptotic cells have certain characteristic morphology, they are often not easy to detect or visualize especially when they display as the early apoptotic cells [12,13]. When a cell undergoes apoptosis, endogenous DNA endonucleases are activated, and the DNA is degraded into fragments of about 180~200 bp or its multiples. The 3′-OH ends are exposed, and thus TUNEL staining can be used to detect the fragmented DNA [14,15]. Our results showed that TUNEL staining in paraffin sections was a reliable method for detecting apoptosis. In addition to chromatin condensation during the early apoptosis process, the nuclei of apoptotic cells become narrowed, and the cytoplasm is condensed after the release of apoptotic bodies. Therefore, TUNEL staining is a more reliable method than morphology observation alone [16]. On the whole, TUNEL staining has the advantages of detecting more apoptotic cells, thus resulting to elevation of the apoptotic index. Therefore it could reflect the extent of apoptosis more accurately, and should be regarded as a simple, fast and efficient method for analysis of tumor apoptosis cells.

It is a controversial issue whether a relationship exists between the apoptosis rate and the degree of malignancy and the prognosis of the patients [17]. Until now it has been generally believed that the higher the apoptosis rate, the slower the tumor growth, thus resulting in a better prognosis [18,19]. We found that the positive apoptosis rate in osteosarcoma by TUNEL staining was 53.6%, which was higher than that of normal tissues. The patients with higher long-term survival had a higher apoptosis rate. In contrast to this study, Lipponen *et al.*[20] determined the cell apoptotic index of bladder cancer and breast cancer according to morphological criteria, and found that poor prognosis correlated with high apoptotic index. Moreover, AI was related significantly to high proliferation rate of cancer cells. Local reports on osteosarcoma had similar results [21]. We analyzed the reasons for this and hypothesized that it could be related to experimental techniques and the different observation phase of apoptosis. If the apoptosis levels of tumor at different stages were different, the results would be different too. The emergence of early apoptotic cells may be positively correlated with tumor cell proliferation, and the emergence of apoptotic bodies at the latter stage could explain the inhibition of tumor growth more exactly. Because the TUNEL technique has high resolution for apoptotic bodies, it is more accurate than the standard morphology analysis.

We presumed that the apoptosis level of tumor tissue was elevated owing to speed up of tumor growth as well as to centrical hypoxia necrosis of larger tumor [22], and the genuine reasons deserved further probing. Elevation of serum ALP of patients with malignant bone tumor often predict more osteogenesis activity of tumor cells, hithreto, ALP is confirmed as a valuable index to assess prognosis and therapeutic results of osteosarcoma [23].

Although the combination of modern surgery and systemic chemotherapy has improved osteosarcoma treatment dramatically, near half of postoperative patients die from pulmonary metastasis of osteosarcoma cells that have poor response to chemotherapy [24]. Deficiency of valid indicators for predicting malignant degree and prognosis of osteosarcoma is attributed to one of reasons of therapeutic failure [25]. Detection of AI is not among the routine pathologic test subjects for tumor specimen, however we believe that testing of AI in biopsy tissue of osteosarcoma before treatment, combining with serum index such as ALP, would help for comprehensive analyze and judgment of malignance and prognosis of osteosarcoma cases, thereby for guidance to chemotherapy and surgical protocol.

## Competing interest

There are no conflicts of interest in our study.

## Authors’ contributions

XW designed research. BC and XW performed research. LMLwas responsible for statistical analysis. XW drafted the manuscript. ZDC revised it critically for important intellectual content. All authors read and approved the final manuscript.
